# Safety and Efficacy of the Supine Position with the Right Shoulder Raised versus the Left Lateral Position in Peroral Endoscopic Myotomy for Achalasia: A Large-Sample Retrospective Study

**DOI:** 10.1155/2022/3202212

**Published:** 2022-09-22

**Authors:** Nanjun Wang, Ningli Chai, Longsong Li, Yawei Bi, Shengzhen Liu, Wengang Zhang, Shasha Wang, Enqiang Linghu

**Affiliations:** Department of Gastroenterology, The First Medical Center of Chinese PLA General Hospital, Beijing, China

## Abstract

**Background:**

The correct surgical position is very important in the treatment of peroral endoscopic myotomy (POEM) for achalasia, which can make the procedure safer and more efficient. Currently, there are two commonly used positions: the supine position with the right shoulder raised and the left lateral position. This study aims to evaluate the differences in the safety and efficacy of these two positions.

**Methods:**

We conducted a retrospective study of 702 patients with achalasia undergoing POEM from December 2010 to December 2020. These patients were divided into the supine position with the right shoulder raised group (*n* = 579) and the left lateral position group (*n* = 123). The efficacy of POEM and adverse events were analyzed.

**Results:**

The clinical characteristics were similar in both groups, and there were no significant differences between the two groups in the Eckardt score change, lower esophageal sphincter (LES) basal pressure or residual pressure after POEM (all *p* > 0.05). The mean operative time in the supine position with the right shoulder raised group was significantly shorter than that in the left lateral position group (43.5 min vs. 54.6 min, respectively, *p* < 0.001). In addition, the differences between the two groups in terms of gas-related complications, such as pneumoperitoneum, pneumomediastinum, and subcutaneous emphysema were statistically significant (all *p* < 0.05).

**Conclusions:**

The efficacy of POEM was comparable between the two groups. However, the supine position with the right shoulder raised significantly reduced the operative time and the rate of procedure-related adverse events, especially gas-related complications.

## 1. Introduction

Achalasia is a functional disease that involves esophageal dynamic dysfunction of unknown cause. It is characterized by aperistalsis of the esophageal body and failure of the lower esophageal sphincter (LES) to relax, with various symptoms, including dysphagia, regurgitation, chest pain, and weight loss [1, 2]. Nowadays, peroral endoscopic myotomy (POEM) is widely used as a safe and efficient therapy for achalasia [3, 4]. The clinical remission rate can be as high as 89–100% [5, 6]. During POEM, correct patient positioning is very important for a safer and easier procedure. At present, the positions commonly used during endoscopy are the left lateral position, supine position, and the supine position with the right shoulder raised. However, there are no reports on which position is the most appropriate for POEM, and endoscopists usually select the position according to their own operating habits.

**Figure 1:**
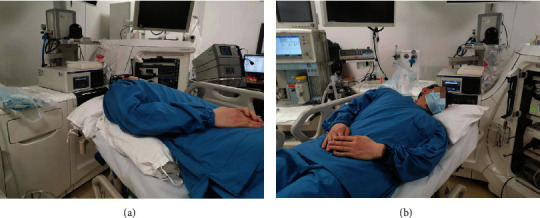
(a) The patient's right shoulder was raised by a cushion. (b) The recommended elevation angle of the right shoulder was about 30°.

The left lateral position is the conventional position for endoscopic diagnosis and treatment, and many endoscopists also routinely use this position for POEM. The supine position facilitates selection of the proximal posterior esophageal wall for surgery; however, due to the twisted degree of the patient's head, it may be difficult to advance the endoscope; thus, this position is rarely used in POEM. In recent years, we have gradually used the supine position with the right shoulder raised to perform POEM. This position falls in between the left lateral and supine position, with the patient's head less twisted. We usually use a cushion or pillow to raise the patient's right shoulder and the recommended elevation angle of the right shoulder is about 30° (Figure 1).

No study has reported the safety and efficacy of using different positions in POEM for the treatment of achalasia. Therefore, we designed this retrospective study to compare the safety and efficacy of POEM in the left lateral position and supine position with the right shoulder raised to promote further development of POEM.

## 2. Methods

### 2.1. Patients

The records of 702 consecutive patients who received POEM to treat achalasia at the First Medical Center of Chinese PLA General Hospital between December 2010 and December 2020 were reviewed in this single-center retrospective study. Before the operation, we explained the relevant procedures in detail to each patient, including possible perioperative complications, treatment plans and postoperative follow-up, which were fully understood by all patients. The study was approved by the Ethics Committee of the Chinese PLA General Hospital, and all patients voluntarily participated in the study and signed written informed consent forms.

### 2.2. Ling Classification

Endoscopic classification of achalasia is defined by the Ling classification, which includes three types [7]: Ling I, dilated esophagus, wall of the esophagus is straight and smooth; Ling II, dilated tortuous esophagus, with a circular or semicircular structure; and Ling III, esophageal cavity dilates, with a diverticular structure. Ling II was further classified into three subtypes: IIa (with a thin circular structure), IIb (with a semicircular structure and the midpoint within one-third of the cavity), and IIc (with a semicircular structure and the midpoint beyond one-third of the cavity). Ling III was also further classified into three subtypes: IIIl (diverticular structure mainly in the left wall of the esophagus), IIIr (diverticular structure mainly in the right wall of the esophagus), and IIIlr (diverticular structure in both the left and right walls of the esophagus).

### 2.3. Indicators Monitored

In this study, the main indicators monitored included the efficacy of POEM, perioperative complications and gas-related adverse events. The efficacy mainly depended on the evaluation of symptomatology, which was concluded by comparing the preoperative and postoperative Eckardt score [8] and high-resolution manometry (HRM) [9]. Perioperative complications mainly included bleeding and perforation, while gas-related adverse events included pneumothorax, pneumoperitoneum, pneumomediastinum, and subcutaneous emphysema.

### 2.4. Instruments and POEM

A gastroscope and a high-frequency generator were used during POEM. A disposable injector with a normal saline solution was used for submucosal injections. The Triangle tip knife was used to establish the submucosal tunnel and myotomy. Hemostatic forceps and clips were used to prevent hemorrhage and perforation. Carbon dioxide gas was used for insufflation with a CO_2_ insufflator during all procedures.

**Figure 2:**
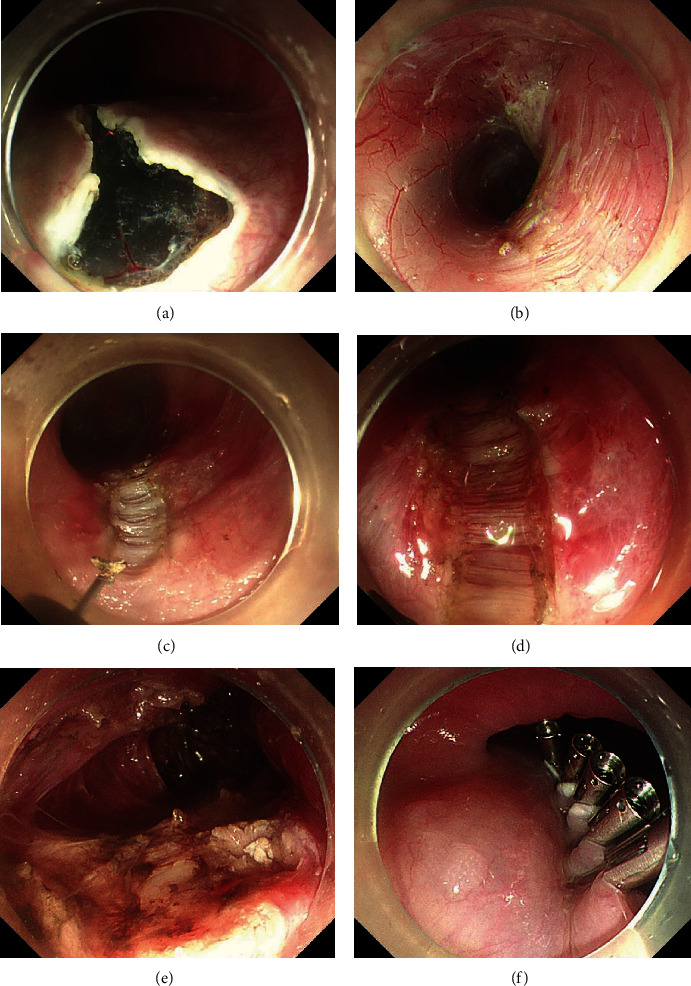
Peroral endoscopic myotomy procedures. (a) A reverse T entry incision was performed. (b) The submucosal tunnel was established. (c) and (d) Partial circular muscle was cut at the starting point of the myotomy. (e) The end of the myotomy. (f) Closure of the tunnel entry with clips.

All patients receiving POEM were required to fast for 48 hours before the operation and complete esophageal cleansing by gastroscopy. The surgical steps in POEM have been reported previously [10], and they include three main steps: establishment of a submucosal tunnel, myotomy, and sealing the tunnel entrance (Figure 2). All procedures were performed by two experienced endoscopists (Enqiang Linghu and Ningli Chai), who are very skilled in operating on patients in two body positions without significant differences. It is worth mentioning that five types of myotomy methods were used, which were an inner circular muscle incision, circular muscle incision combined with balloon shaping, glasses-type muscle incision, progressive full-thickness myotomy, and full-thickness muscle incision [11]. After the operation, all patients were required to stay in bed and fasted for 3 days. Proton pump inhibitors (PPIs) were administered intravenously and antibiotics were used prophylactically to avoid infection. In addition, if perioperative bleeding, perforation, and other complications occurred, the patients received active symptomatic treatment such as drug hemostasis, endoscopic hemostasis, and endoscopic closure of the perforation, as well as interventional embolization for hemostasis and surgery. Other treatments were also administered when necessary. Patients were followed up at 3 months, 6 months, and 1 year after POEM, including endoscopy, 24-hour esophageal pH monitoring, and HRM. Thereafter, annual follow-up was strongly recommended.

### 2.5. Statistical Analysis

For the data related to this study, we designated special personnel to record, manage, and assist with statistical analysis. Measurement data are expressed as the mean value ± standard deviation or median with range, whereas numerical data are described by frequency and percentage and were compared using the *χ*^2^ or Fisher's exact test. The measurement data were analyzed by *t*-test and one-way analysis of variance or rank-sum test according to whether the data conformed to a normal distribution. Chi-square tests were used to compare categorical variables. Multiple regression analysis or logistics regression analysis is used to explore the relationship between multiple variables. *p* < 0.05 was considered statistically significant.

## 3. Results

### 3.1. Clinical Characteristics of the Two Groups

A total of 702 patients were enrolled in the study, including 309 males and 393 females, aged from 18 to 85 years (mean 44.6 years). 579 patients were placed in the supine position with the right shoulder raised, while the other 123 patients were placed in the left lateral position. The average disease course of the patients in the supine position with the right shoulder raised group was 7.1 years (range 1 month to 45 years) and that in the left lateral position group was 5.4 years (range 2 months to 33 years). Before undergoing POEM, 112 patients (19.3%) in the supine position with the right shoulder raised group had received previous treatments (57 treated with balloon dilation, 33 with Botox injection, 3 with temporary stenting, 8 with Heller myotomy, 3 with balloon dilation + Botox injection, 1 with balloon dilation + temporary stenting, 1 with Botox injection + temporary stenting, 1 with temporary stenting + Heller myotomy, and 5 POEM), whereas 34 patients (27.6%) in the left lateral group had received previous treatments (20 with balloon dilation, 10 with Botox injection, 2 with temporary stenting, 1 with temporary stenting + Heller myotomy, and 1 with POEM). The Ling classification in the supine position with the right shoulder raised group included 69 Ling type I patients, 485 Ling type II patients, and 25 Ling type III patients; the Ling classification in the left lateral group included 23 Ling type I patients, 92 Ling type II patients, and 8 Ling type III patients, respectively. Chicago II was the most common type of achalasia in both groups (73.9% vs. 73.2%). There were no statistically significant differences between the two groups in terms of gender, age, duration of symptoms, previous treatment, Ling classification or Chicago classification (all *p* > 0.05). The clinical characteristics of the two groups are shown in Table 1.

### 3.2. Comparison of POEM-Related Parameters

All patients in both groups successfully underwent POEM, and the detailed data are shown in Table 2. In the supine position with the right shoulder raised group, the mean lengths of the tunnel and myotomy were 10.6 cm (4–20 cm) and 7.0 cm (3–15 cm), respectively, while those in the left lateral position group were 11.9 cm (7–26 cm) (*p* = 0.297), and 6.6 cm (2–23 cm) (*p* = 0.103), respectively. Progressive full-thickness myotomy was performed in 85.3% (494 cases) of patients in the supine position with the right shoulder raised group and in 75.6% (93 cases) of patients in the left lateral position group. The types of myotomy in the remaining patients in the two groups included inner circular muscle incision (14 vs. 4 cases), circular muscle incision combined with balloon shaping (19 vs. 6 cases), glasses-type muscle incision (14 vs. 5 cases), and full-thickness muscle incision (38 vs. 15 cases). Although the types of myotomy differed among the patients, there were no statistically significant differences between the two groups (*p* = 0.087). However, the mean operative time in the supine position with the right shoulder raised group [43.5 min (range 17–180 min)] was significantly shorter than that in the left lateral position group [54.6 min (range 22–170 min)] (*p* < 0.001).

### 3.3. Symptom Relief and HRM Outcomes

As shown in Table 3, 532 patients (91.9%) in the supine position with the right shoulder raised group received a symptom score during follow-up, with a mean follow-up time of 23.5 months (3–60 months), while the follow-up time in the left lateral position group was 25.4 months (3–66 months). Based on a postoperative Eckardt score of ≤3, which was defined as successful treatment, there was no significant difference in the therapeutic success between the two groups (96.8% vs. 95.3%, *p* = 0.394). Both groups of patients showed a significant improvement in the post-treatment Eckardt score. However, there was no statistically significant difference in the Eckardt score between the two groups before and after POEM [5.8 (range 0–12) vs. 5.8 (range 0–10)] (*p* = 0.850).

During follow-up, postoperative gastroesophageal reflux occurred in 83 patients, including 68 in the supine position with the right shoulder raised group and 15 in the left lateral position group. There was no statistically significant difference in the incidence of reflux between the two groups (*p* = 0.728). All patients had effective relief of symptoms following oral administration of PPIs. Due to the obvious improvement in clinical symptoms and discomfort during manometry, 182 patients in the supine position with the right shoulder raised group and 31 patients in the left lateral position group completed postoperative manometry, respectively. The basal and residual pressure of the LES in both groups decreased significantly after POEM; however, the difference in the degree of decreased residual pressure of the LES between the two groups was not statistically significant (*p* = 0.105, *p* = 0.086).

### 3.4. Comparison of POEM-Related Adverse Events

Adverse events occurred in 53 (9.2%) patients in the supine position with the right shoulder raised group and in 26 (21.1%) patients in the left lateral position group, representing a significant difference (*p* < 0.001). In the supine position with the right shoulder raised group, 9 (1.6%) patients developed mucosal injury and 6 (1.0%) patients developed pneumothorax, compared with 2 (1.6%) and 2 (1.6%) patients in the left lateral position group, respectively, and the difference between the two groups was not statistically significant (*p* = 1.000, *p* = 0.927). Mucosal injury was closed with tissue clips or porcine fibrin glue. One patient with serious pneumothorax in the left lateral position group, whose right lung was 80% compressed, was instantly relieved after exhausting approximately 1400 ml of gas, and the remaining patients with mild pneumothorax gradually recovered spontaneously. However, the incidence of pneumoperitoneum, pneumomediastinum, and subcutaneous emphysema was 2.8%, 0.3%, and 3.4%, respectively, in the supine position with the right shoulder raised group and 7.3%, 2.4%, and 8.1%, respectively, in the left lateral position group, and the differences were statistically significant between the two groups (*p* = 0.027, *p* = 0.040, *p* = 0.020). All patients with pneumoperitoneum were treated by abdominocentesis with a 10 ml syringe, and 4 of the 10 patients with subcutaneous emphysema in the left lateral position group required puncture decompression. The remaining patients with pneumomediastinum and subcutaneous emphysema did not require specific clinical interventions. No massive hemorrhage occurred during the procedure, and no delayed bleeding occurred in either group.

In addition, 14 (2.4%) patients in the supine position with the right shoulder raised group and 2 (1.6%) patients in the left lateral position group developed fever after POEM, but the difference between the two groups was not statistically significant (*p* = 1.000). Among them, 1 patient in the left lateral position group experienced bacteremia due to *Propionibacterium acnes*, and this patient's temperature gradually returned to normal after taking third-generation cephalosporin. The POEM-related adverse events are also listed in Table 2.

### 3.5. Multivariate Regression Analysis and Logistics Regression Analysis

The results of multivariate regression analysis and logistics regression analysis that affect the operative time and occurrence of gas-related complications are shown in Tables 4 and 5. Multivariate regression analysis showed that the longer disease duration, left lateral position, longer tunnel length, and full-thickness myotomy were associated with longer operative time, with statistically significant differences (*p* = 0.006, 0.004, 0.003, and 0.003, respectively). However, in logistics regression analysis, only the left lateral position was more prone to gas-related complications (*p* = 0.009), while the course of disease, tunnel length, and whether full-thickness myotomy was performed did not present correlation with gas-related complications for the time being (*p* = 0.555, 0.105, 0.577, respectively).

## 4. Discussion

Since the Japanese scholars Inoue et al. first reported the POEM used to treat achalasia in 2010 [10], research on POEM has continuously increased [12–17]. Some of these studies are conducive to the further improvement and development of POEM, and our exploration of the operative position is based on this intention. Many endoscopists use the conventional left lateral position to perform POEM. However, in our clinical practice and investigations, we have found that the supine position with the right shoulder raised seems to be more advantageous in some aspects. Therefore, we designed this study to clarify the influence of surgical position on POEM.

It should be noted that we also initially used the supine position to perform POEM. However, we only completed this in a few cases as the patient's head was too twisted, the endoscopic propulsion was difficult, and fluids remained in the rear esophageal cavity due to gravity, which might soak the tunnel incision during the procedure and affect the endoscopic field. Therefore, the purpose of this study was to compare the difference between the conventional left lateral position and the supine position with the right shoulder raised which is increasingly being used clinically.

In our study, 579 patients were enrolled in the supine position with the right shoulder raised group and 123 in the left lateral position group, respectively. There were no significant differences between the two groups in clinical baseline data, such as age, sex, disease course, previous treatment history, Ling classification, and Chicago subtype of achalasia. In the comparison of procedure-related parameters, there was no significant difference in tunnel length, myotomy length (including esophageal myotomy length, gastric myotomy length, and total myotomy length), and types of myotomy between the two groups. However, the operating time in the supine position with the right shoulder raised group was significantly shorter than that in the left lateral position group (*p* < 0.001). Based on this, we performed further multivariate regression analyses for several factors that may influence the operative time, and the results showed that prolonged disease duration, long intraoperative tunnel construction, and full-thickness myotomy, and left lateral position increased the operative time. In general, patients with a longer course of disease may have more pronounced dilatation and distortion of the esophagus and higher pressure of LES, which may require more complex surgical procedures and increase the operative time. Similarly, building a longer tunnel and performing a full-thickness myotomy during the operation also complicates the operation and naturally takes more time. The influence of the above factors on the operative time is relatively easy to understand, while the influence of different operative positions has not been reported before, which is also the focus of our attention.

The left lateral position is the routine position for endoscopic examinations, which helps the endoscopist to identify the anatomic orientation of the esophageal wall because the common direction to operate a device under endoscopy is the 6 o'clock position; however, it is necessary to rotate the endoscope to adjust the correct direction for a tunnel to be established in the right rear esophageal wall. By contrast, the supine position with the right shoulder raised retains the advantages of the supine position in favor of the selection of the proximal posterior esophageal wall for surgery, while the patient's head is less twisted so that the device can be withdrawn in a naturally relaxed way to the proximal rear esophageal wall under endoscopy, facilitating the approach and withdrawal of the endoscope as well as the whole operation. In addition, as reported in the relevant literature [18], the supine position with the right shoulder raised is advantageous with respect to no fluid retention at the right rear esophageal wall (because it is not the lowest point in this position), with no effect on the surgical field. All of these factors contribute to the faster completion of POEM in the supine position with the right shoulder raised.

POEM-related adverse events reported in previous studies were also the focus of our attention [19–21]. Differences in gas-related complications were observed between the two groups in the present study. By comparing the distribution differences between two groups and conducting further logistics regression analysis, the results showed that the left lateral position group was more prone to gas-related complications than the supine position with the right shoulder raised group. Specifically, the incidence of pneumoperitoneum, pneumomediastinum, and subcutaneous emphysema was significantly lower in the supine position with the right shoulder raised group than in the left lateral position group. In terms of pneumothorax, although the difference between the two groups was not statistically significant, the incidence was higher in the left lateral position group, and 1 patient with severe pneumothorax and 4 patients with subcutaneous emphysema requiring puncture decompression were all in the left lateral position group. Overall, the supine position with the right shoulder raised was superior in controlling gas-related complications. Anatomically, the esophagus is located behind the trachea and heart and in front of the spine. It is relatively safe to establish a submucosal tunnel at the proximal posterior wall of the esophagus away from major organs. This also coincides with the direction in which the supine position with the right shoulder raised establishes the tunnel. On the other hand, in the left lateral position group, the operation required more time, and the gas had more time to diffuse through the tunnel cavity to the outer esophageal space before the tunnel reached below the relative plane of the diaphragm. These were the two main reasons for the higher incidence of gas-related complications in the left lateral position group.

In addition to gas-related complications, intraoperative mucosal injury and postoperative fever occurred in a small number of patients. The differences in these complications were not significant between the two groups. However, it is worth noting that 1 patient with postoperative fever developed bacteremia in the left lateral position group. During POEM, the esophageal cavity is not completely sterile, and some small blood vessels are inevitably exposed in the process of establishing the submucosal tunnel and myotomy, which creates conditions for bacteria to enter the blood. As mentioned above, the left lateral position group had a longer operation duration, which increased the potential risk of bacteremia. This also demonstrates another advantage of the supine position with the right shoulder raised.

During follow-up, treatment efficacy was satisfactory in both groups, with symptom relief rates reaching over 95%, and there was no significant difference between the groups. Postoperative follow-up HRM data showed that the pressure of the LES (including basal pressure and residual pressure) in both groups was significantly relieved compared with that before surgery, which was also one of the manifestations showing the efficacy of POEM. These results were similar to those in previous studies [5, 6]. We also noted that a small number of patients developed reflux after POEM. Some of these patients had higher Eckard scores due to the discomfort caused by reflux, despite the fact that their dysphagia symptoms were generally relieved. This suggests that further research on the effective control of reflux after POEM may be needed in the future.

The present study had several limitations. One limitation was its retrospective design and potential selection bias, as our hospital is a tertiary referral center. Other limitations of the study included a lack of HRM, 24-hour pH testing, and timed-barium swallow after POEM in some patients. Therefore, prospective multicenter, randomized clinical trials with long-term follow-up periods should be carried out in the future.

## 5. Conclusion

POEM is a safe and efficient treatment for patients with achalasia, irrespective of whether it is performed in the supine position with the right shoulder raised or left lateral position. Therapeutic success was achieved in 96.6% of cases. No significant differences between the two groups were observed in terms of the changes in the Eckardt score, LES basal pressure or residual pressure after POEM. Compared to the left lateral position group, the supine position with the right shoulder raised group had a shorter operating time and fewer procedure-related adverse events, especially gas-related complications.

## Figures and Tables

**Table 1:** Clinical characteristics of the two groups.

	Supine position with the right shoulder raised group (*n* = 579)	Left lateral position group (*n* = 123)	*p* value
Sex, male/female (*n*)	249/330	60/63	0.241
Age, mean (range) (years)	45.7 (18–85)	43.3 (19–77)	0.088
Duration of symptoms, mean (range) (years)	7.1 (0.1–45)	5.4 (0.2–33)	0.069
Previous treatment [*n* (%)]	112 (19.3)	34 (27.6)	0.636
Balloon dilation	57 (9.8)	20 (16.3)	—
Botox injection	33 (5.7)	10 (8.1)	—
Temporary stenting	3 (0.5)	2 (1.6)	—
Heller myotomy	8 (1.4)	0 (0)	—
Balloon dilation + Botox injection	3 (0.5)	0 (0)	—
Balloon dilation + temporary stenting	1 (0.2)	0 (0)	—
Botox injection + temporary stenting	1 (0.2)	0 (0)	—
Temporary stenting + Heller myotomy	1 (0.2)	1 (0.8)	—
Peroral endoscopic myotomy	5 (0.9)	1 (0.8)	—
Ling classification [*n* (%)]			0.116
I	69 (11.9)	23 (18.7)	—
IIa	157 (27.1)	32 (26.0)	—
IIb	148 (25.6)	32 (26.0)	—
IIc	180 (31.1)	28 (22.8)	—
IIIl	8 (1.4)	5 (4.1)	—
IIIr	7 (1.2)	1 (0.8)	—
IIIlr	10 (1.7)	2 (1.6)	—
Chicago subtype of achalasia [*n* (%)]			0.877
I	113 (19.5)	26 (21.1)	—
II	428 (73.9)	90 (73.2)	—
III	38 (6.6)	7 (5.7)	—

**Table 2:** Comparisons of POEM-related parameters and adverse events between the two groups.

	Supine position with the right shoulder raised group (*n* = 579)	Left lateral position group (*n* = 123)	*p* value
Types of myotomy [*n* (%)]	—	—	0.087
Inner circular muscle incision	14 (2.4)	4 (3.2)	—
Circular muscle incision + balloon shaping	19 (3.3)	6 (4.9)	—
Glasses-type muscle incision	14 (2.4)	5 (4.1)	—
Progressive full-thickness myotomy	494 (85.3)	93 (75.6)	—
Full-thickness muscle incision	38 (6.6)	15 (12.2)	—
Operating time, mean (range) (min)	43.5 (17–180)	54.6 (22–170)	<0.001
Tunnel length, mean (range) (cm)	10.6 (4–20)	11.9 (7–26)	0.297
Myotomy length, mean (range) (cm)	—	—	—
Esophageal	5.0 (0–13)	4.7 (0–21)	0.108
Gastric	2.0 (0–4)	1.9 (0–3)	0.937
Total	7.0 (3–15)	6.6 (2–23)	0.103
All intraoperative adverse events [*n* (%)]	53 (9.2)	26 (21.1)	<0.001
Mucosal injury	9 (1.6)	2 (1.6)	1.000
Pneumothorax	6 (1.0)	2 (1.6)	0.927
Pneumoperitoneum	16 (2.8)	9 (7.3)	0.027
Pneumomediastinum	2 (0.3)	3 (2.4)	0.040
Subcutaneous emphysema	20 (3.4)	10 (8.1)	0.020
Fever (temperature > 38.0°C) [*n* (%)]	14 (2.4)	2 (1.6)	1.000

**Table 3:** Comparisons of Eckardt scores and HRM between the two groups.

	Supine position with the right shoulder raised group (*n* = 579)	Left lateral position group (*n* = 123)	*p* value
Follow-up period, mean (range) (months)	23.5 (3–60)	25.4 (3–66)	0.106
Symptom score follow-up rate [*n* (%)]	532 (91.9)	107 (87.0)	0.085
Treatment success (Eckardt score ≤3) [*n* (%)]	515 (96.8)	102 (95.3)	0.394
Eckardt score, mean (range)	—	—	—
Pre-treatment	7.0 (4–12)	7.2 (4–12)	0.266
Post-treatment	1.2 (0–4)	1.4 (0–5)	0.092
Pre-post	5.8 (2–10)	5.8 (2–10)	0.850
Gastroesophageal reflux [*n* (%)]	68 (12.8)	15 (14.0)	0.728
HRM follow-up rate [*n* (%)]	182 (31.4)	31 (25.2)	0.172
LES basal pressure, mean (range) (mm Hg)	—	—	—
Pre-treatment	37.6 (0.7–100.6)	35.6 (6.2–73.9)	0.396
Post-treatment	15.6 (0.6–56.2)	17.2 (0.5–52.7)	0.247
Pre-post	22.0 (–9.4 to 79.1)	18.4 (–4.9 to 46.5)	0.105
LES residual pressure, mean (range) (mm Hg)	—	—	—
Pre-treatment	29.1 (1–83.2)	27.3 (6.5–74.2)	0.318
Post-treatment	11.1 (0.7–28.9)	12.1 (0.5–36.2)	0.266
Pre-post	18.0 (–5 to 64)	15.1 (–4.3 to 47.7)	0.086

**Table 4:** Multivariate regression analysis of operative time on course of disease, whether full-thickness myotomy, tunnel length and operative position.

	*B*	Beta	*F*	*R* ^2^ (adjusted *R*^2^)	*t*	*p*
Constant	36.515	—	13.320∗∗∗	0.071 (0.066)	5.403	<0.001
Disease duration	0.278	0.102			2.770	0.006
Full-thickness myotomy	−6.472	−0.123			−2.964	0.003
Tunnel length	1.036	0.112			3.008	0.003
Operative position	6.480	0.123			2.922	0.004

Notes: ∗*p* < 0.05; ∗∗*p* < 0.01; ∗∗∗*p* < 0.001.

**Table 5:** Binary logistic regression analysis of the occurrence of gas-related complications on course of disease, whether full-thickness myotomy, tunnel length and operative position.

	*B*	SE	Wald	*v*	*p*	Exp(*B*)	95% CI of Exp(*B*)	
							Lower limit	Upper limit
Constant	−4.694	1.211	15.016	1	<0.001	0.009	—	—
Disease duration	0.012	0.020	0.348	1	0.555	1.012	0.973	1.051
Full-thickness myotomy	−0.214	0.383	0.312	1	0.577	0.807	0.381	1.711
Tunnel length	0.108	0.067	2.629	1	0.105	1.114	0.978	1.270
Operative position	0.966	0.369	6.874	1	0.009	2.629	1.276	5.413

## Data Availability

All data obtained or analyzed during this work are included within the article.

## Abbreviations

POEM: Peroral endoscopic myotomy
LES: Lower esophageal sphincter
HRM: High-resolution manometry
PPI: Proton pump inhibitor.
